# Assessing Information Transmission in Data Transformations with the Channel Multivariate Entropy Triangle

**DOI:** 10.3390/e20070498

**Published:** 2018-06-27

**Authors:** Francisco J. Valverde-Albacete, Carmen Peláez-Moreno

**Affiliations:** Department of Signal Theory and Communications, Universidad Carlos III de Madrid, Leganés 28911, Spain

**Keywords:** entropy, entropy visualization, entropy balance equation, Shannon-type relations, multivariate analysis, machine learning evaluation, data transformation

## Abstract

Data transformation, e.g., feature transformation and selection, is an integral part of any machine learning procedure. In this paper, we introduce an information-theoretic model and tools to assess the quality of data transformations in machine learning tasks. In an unsupervised fashion, we analyze the transformation of a discrete, multivariate source of information $\bar{X}$ into a discrete, multivariate sink of information $\bar{Y}$ related by a distribution $P_{\bar{X}\bar{Y}}$. The first contribution is a decomposition of the maximal potential entropy of $\left(\bar{X},\bar{Y}\right)$, which we call a balance equation, into its (a) non-transferable, (b) transferable, but not transferred, and (c) transferred parts. Such balance equations can be represented in (de Finetti) entropy diagrams, our second set of contributions. The most important of these, the aggregate channel multivariate entropy triangle, is a visual exploratory tool to assess the effectiveness of multivariate data transformations in transferring information from input to output variables. We also show how these decomposition and balance equations also apply to the entropies of $\bar{X}$ and $\bar{Y}$, respectively, and generate entropy triangles for them. As an example, we present the application of these tools to the assessment of information transfer efficiency for Principal Component Analysis and Independent Component Analysis as unsupervised feature transformation and selection procedures in supervised classification tasks.

## 1. Introduction

Information-related considerations are often cursorily invoked in many machine learning applications, sometimes to suggest why a system or procedure is seemingly better than another at a particular task. In this paper, we set out to ground our work on measurable evidence phrases such as “this transformation retains more information from the data” or “this learning method uses the information from the data better than this other”.

This has become particularly relevant with the increase of complexity of machine learning methods, such as deep neuronal architectures [1], which prevents straightforward interpretations. Nowadays, these learning schemes almost always become black-boxes, where the researchers try to optimize a prescribed performance metric without looking inside. However, there is a need to assess what the deep layers are actually accomplishing. Although some answers have started to appear [2,3], the issue is by no means settled.

**Figure 1:**
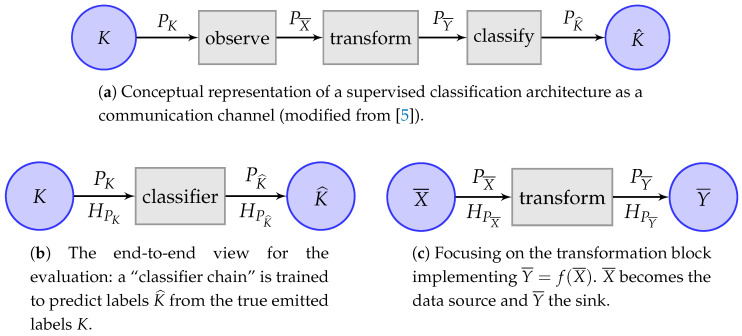
Different views of a supervised classification task as an information channel: (**a**) as individualized blocks; (**b**) for end-to-end evaluation; and (**c**) focused on the transformation.

In this paper, we put forward that framing the previous problem in a generic information-theoretical model can shed light on it by exploiting the versatility of information theory. For instance, a classical end-to-end example of an information-based model evaluation can be observed in Figure 1a. In this supervised scheme introduced in [4], the evaluation of the performance of the classifier involves only the comparison of the true labels *K* vs. the predicted labels $\hat{K}$. This means that all the complexity enclosed in the classifier box cannot be accessed, measured or interpreted.

In this paper, we want to expand the previous model into the scheme of Figure 1a, which provides a more detailed picture of the contents of the black-box where:

- A random source of classification labels *K* is subjected to a measurement process that returns random observations $\bar{X}$. The *n* instances of pairs $(k_{i},{\bar{x}}_{i}),1\leq i\leq n$ is often called the (task) dataset.
- Then, a generic data transformation block may transform the available data, e.g., the observations in the dataset $\bar{X}$, into other data with “better” characteristics, the transformed feature vectors $\bar{Y}$. These characteristics may be representational power, independence among individual dimensions, reduction of complexity offered to a classifier, etc. The process is normally called feature transformation and selection.
- Finally, the $\bar{Y}$ are the inputs to an actual classifier of choice that obtains the predicted labels $\hat{K}$.

This would allow us to better understand the flow of information in the classification process with a view toward assessing and improving it.

Note the similarity between the classical setting of Figure 1b and the transformation block of Figure 1a reproduced in Figure 1c for convenience. Despite this, the former represents a Single-Input Single-Output (SISO) block with $\left(K,\hat{K}\right)∼P_{K\hat{K}}$, whereas the latter represents a multivariate Multiple-Input Multiple-Output (MIMO) block described by the joint distribution of random vectors $\left(\bar{X},\bar{Y}\right)∼P_{\bar{X}\bar{Y}}$.

This MIMO kind of block may represent an unsupervised transformation method—for instance, a Principal Component Analysis (PCA) or Independent Component Analysis (ICA)—in which case, the “effectiveness” of the transformation is supplied by a heuristic principle, e.g., least reconstruction error on some test data, maximum mutual information, etc. However, it may also represent a supervised transformation method—for instance, $\bar{X}$ are the feature instances, and $\bar{Y}$ are the (multi-)labels or classes in a classification task, or $\bar{Y}$ may be the activation signals of a convolutional neural network trained using an implicit target signal— in which case, the “effectiveness” should measure the conformance to the supervisory signal.

In [4], we argued for carrying out the evaluation of classification tasks that can be modeled by Figure 1b with the new framework of entropy balance equations and their related entropy triangles [4,5,6]. This has provided a means of quantifying and visualizing the end-to-end information transfer for SISO architectures. The gist of this framework is explained in Section 2.1: if a classifier working on a certain dataset obtained a confusion matrix $P_{K\hat{K}}$, then we can information-theoretically assess the classifier by analyzing the entropies and information in the related distribution $P_{K\hat{K}}$ with the help of a balance equation [6]. However, looking inside the black-box poses a challenge since $\bar{X}$ and $\bar{Y}$  are random vectors and most information-theoretic quantities are not readily available in their multivariate version.

If we want to extend the same framework of evaluation to random vectors in general, we need the multivariate generalizations of the information-theoretic measures involved in the balance equations, an issue that is not free of contention. With this purpose in mind, we review the best-known multivariate generalizations of mutual information in Section 2.2.

We present our contributions finally in Section 3. As a first result, we develop a balance equation for the joint distribution $P_{\bar{X}\bar{Y}}$ and related representation in Section 3.1 and Section 3.2, respectively. However we are also able to obtain split equations for the input and output multivariate sources only tied by one multivariate extension of mutual information, much as in the SISO case. As an instance of use, in Section 3.3, we analyze the transfer of information in PCA and ICA transformations applied to some well-known UCI datasets. We conclude with a discussion of the tools in light of this application in Section 3.4.

## 2. Methods

In Section 3, we will build a solution to our problem by finding the minimum common multiple, so to speak, of our previous solutions to the SISO block we describe in Section 2.1 and the multivariate source cases, to be described in Section 2.2.

### 2.1. The Channel Bivariate Entropy Balance Equation and Triangle

**Figure 2:**
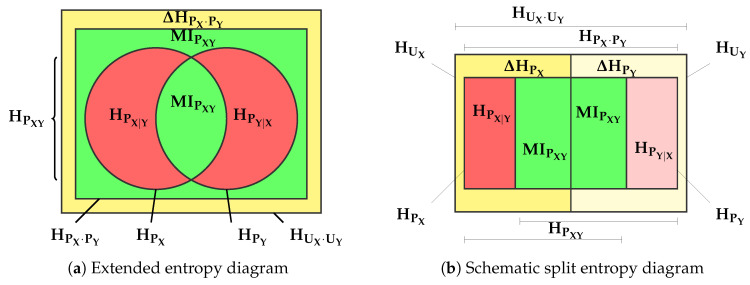
Extended entropy diagram related to a bivariate distribution, from [4].

A solution to conceptualizing and visualizing the transmission of information through a channel where input and output are reduced to a single variable, that is with $|\bar{X}|=1$ and $|\bar{Y}|=1$, was presented in [6] and later extended in [4]. For this case, we use simply *X* and *Y* to describe the random variables. Notice that in the Introduction, and later in the example application, these are called *K* and $\hat{K}$, but here, we want to present this case as a simpler version of the one we set out to solve in this paper. Figure 2a, then, depicts a classical information-diagram (i-diagram) [7,8] of an entropy decomposition around $P_{XY}$ in which we have included the exterior boundaries arising from the entropy balance equation, as we will show later. Three crucial regions can be observed:

- The (normalized) redundancy ([9], Section 2.4), or divergence with respect to uniformity (yellow area), $\Delta H_{P_{X}\cdot P_{Y}}$, between the joint distribution where $P_{X}$ and $P_{Y}$ are independent and the uniform distributions with the same cardinality of events as $P_{X}$ and $P_{Y}$,
  (1) $$\begin{array}{c} \Delta H_{P_{X}\cdot P_{Y}}=H_{U_{X}\cdot U_{Y}}-H_{P_{X}\cdot P_{Y}}. \end{array}$$
- The mutual information, $MI_{P_{XY}}$ [10] (each of the green areas), quantifies the force of the stochastic binding between $P_{X}$ and $P_{Y}$, “towards the outside” in Figure 2a,
  (2) $$\begin{array}{c} MI_{P_{XY}}=H_{P_{X}\cdot P_{Y}}-H_{P_{XY}} \end{array}$$
  but also “towards the inside”,
  (3) $$\begin{array}{c} MI_{P_{XY}}=H_{P_{X}}-H_{P_{X|Y}}=H_{P_{Y}}-H_{P_{Y|X}}. \end{array}$$
- The variation of information (the sum of the red areas), $VI_{P_{XY}}$ [11], embodies the residual entropy, not used in binding the variables,
  (4) $$\begin{array}{c} VI_{P_{XY}}=H_{P_{X|Y}}+H_{P_{Y|X}}. \end{array}$$

Then, we may write the following entropy balance equation between the entropies of *X* and *Y*:

(5) $$\begin{array}{cc} H_{U_{X}\cdot U_{Y}} & =\Delta H_{P_{X}\cdot P_{Y}}+2\cdot MI_{P_{XY}}+VI_{P_{XY}}\\ 0 & \leq \Delta H_{P_{X}\cdot P_{Y}},MI_{P_{XY}},VI_{P_{XY}}\leq H_{U_{X}\cdot U_{Y}} \end{array}$$

where the bounds are easily obtained from distributional considerations [6]. If we normalize (5) by the overall entropy $H_{U_{X}\cdot U_{Y}}$, we obtain:

(6) $$\begin{array}{cccc} 1 & =\Delta^{\prime }H_{P_{X}\cdot P_{Y}}+2\cdot MI_{P_{XY}}^{\prime }+VI_{P_{XY}}^{\prime } & 0 & \leq \Delta^{\prime }H_{P_{X}\cdot P_{Y}},MI_{P_{XY}}^{\prime },VI_{P_{XY}}^{\prime }\leq 1 \end{array}$$

**Figure 3:**
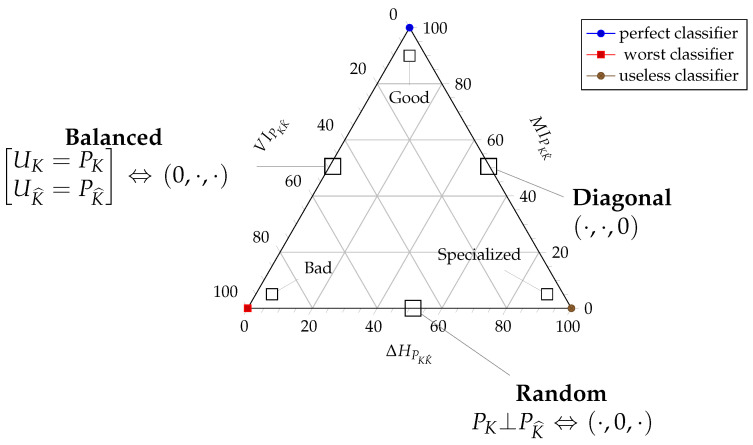
Schematic CBET as applied to supervised classifier assessment. An actual triangle shows dots for each classifier (or its split coordinates see Figure 6 for example) and none of the callouts for specific types of classifiers (from [4]). The callouts situated in the center of the sides of the triangle apply to the whole side.

Equation (6) is the 2-simplex in normalized $\Delta {H^{\prime }}_{P_{X}\cdot P_{Y}}\times 2{MI{\;}^{\prime }}_{P_{XY}}\times {VI{\;}^{\prime }}_{P_{XY}}$ space. Each joint distribution $P_{XY}$ can be characterized by its joint entropy fractions, $F\left(P_{XY}\right)=\left[\Delta H_{P_{XY}}^{\prime },2\cdot MI{\;}_{P_{XY}}^{\prime },VI{\;}_{P_{XY}}^{\prime }\right]$, whose projection onto the plane with director vector (1,1,1) is its de Finetti or compositional diagram [12]. This diagram of the 2-simplex is an equilateral triangle, the coordinates of which are $F(P_{XY})$, so every bivariate distribution is shown as a point in the triangle, and each zone in the triangle is indicative of the characteristics of distributions, the coordinates of which fall in it. This is what we call the Channel Bivariate Entropy Triangle (CBET) whose schematic is shown in Figure 3.

We can actually decompose (5) and the quantities in it into two split balance equations,

(7) $$\begin{array}{cccc} H_{U_{X}} & =\Delta H_{P_{X}}+{MI}_{P_{XY}}+H_{P_{X|Y}} & \;H_{U_{Y}} & =\Delta H_{P_{Y}}+{MI}_{P_{XY}}+H_{P_{Y|X}}. \end{array}$$

with the obvious limits. These can be each normalized by $H_{U_{X}}$, respectively $H_{U_{Y}}$, leading to the 2-simplex equations:

(8) $$\begin{array}{cccc} 1 & =\Delta^{\prime }H_{P_{X}}+{MI^{\prime }}_{P_{XY}}+H_{P_{X|Y}}^{\prime } & \;1 & =\Delta^{\prime }H_{P_{Y}}+{MI}_{P_{XY}}^{\prime }+H_{P_{Y|X}}^{\prime }. \end{array}$$

Since these are also equations on a 2-simplex, we can actually represent the coordinates $F_{X}\left(P_{XY}\right)=\left[\Delta H_{P_{X}}^{\prime },MI{\;}_{P_{XY}}^{\prime },H_{P_{X|Y}}^{\prime }\right]$ and $F_{Y}\left(P_{XY}\right)=\left[\Delta H_{P_{Y}}^{\prime },MI{\;}_{P_{XY}}^{\prime },H_{P_{Y|X}}^{\prime }\right]$ in the same triangle side by side the original $F(P_{XY})$, whereby the representation seems to split in two.

#### Application: The Evaluation of Multiclass Classification

The CBET can be used to visualize the performance of supervised classifiers in a straightforward manner as announced in the Introduction: Consider the confusion matrix $N_{K\hat{K}}$ of a classifier chain on a supervised classification task given the random variable of true class labels $K∼P_{K}$ and that of predicted labels $\hat{K}∼P_{\hat{K}}$ as depicted in Figure 1a, which now play the role of $P_{X}$ and $P_{Y}$. From this confusion matrix, we can estimate the joint distribution $P_{K\hat{K}}$ between the random variables, so that the entropy triangle for $P_{K\hat{K}}$ produces valuable information about the actual classifier used to solve the task [6,13] and even the theoretical limits of the task; for instance, whether it can be solved in a trustworthy manner by classification technology and with what effectiveness.

The CBET acts, in this case, as an exploratory data analysis tool for visual assessment, as shown in Figure 3.

The success of this approach in the bivariate, supervised classification case is a strong hint that the multivariate extension will likewise be useful for other machine learning tasks. See [4] for a thorough explanation of this procedure.

### 2.2. Quantities around the Multivariate Mutual Information

The main hurdle for a multivariate extension of the balance Equation (5) and the CBET is the multivariate generalization of binary mutual information, since it quantifies the information transport from input to output in the bivariate case and is also crucial for the decoupling of (5) into the split balance Equation (7). For this reason, we next review the different “flavors” of information measures describing sets of more than two variables looking for these two properties. We start from very basic definitions both in the interest of self-containment and to provide a script of the process of developing future analogues for other information measures.

To fix notation, let $\bar{X}=\left\{X_{i}|1\leq i\leq m\right\}$ be a set of discrete random variables with joint multivariate distribution $P_{\bar{X}}=P_{X_{1}\ldots X_{m}}$ and the corresponding marginals $P_{X_{i}}\left(x_{i}\right)=\sum_{j\neq i}P_{\bar{X}}\left(\bar{x}\right)$ where $\bar{x}=x_{1}\ldots x_{m}$ is a tuple of *m* elements; likewise for $\bar{Y}=\left\{Y_{j}|1\leq j\leq l\right\}$, with $P_{\bar{Y}}=P_{Y_{1}\ldots Y_{l}}$ and the marginals $P_{Y_{j}}$. Furthermore, let $P_{\bar{X}\bar{Y}}$ be the joint distribution of the (m+l)-length tuples $\bar{X}\bar{Y}$. Note that two different situations can be clearly distinguished:

Situation 1: All the random variables form part of the same set $\bar{X}$, and we are looking at information transfer within this set, or
Situation 2: They are partitioned into two different sets $\bar{X}$ and $\bar{Y}$, and we are looking at information transfer between these sets.

An up-to-date review of multivariate information measures in both situations is [14], which follows the interesting methodological point from [15] of calling information those measures that involve amounts of entropy shared by multiple variables and entropies those that do not—although, this poses a conundrum for the entropy written as the self-information $H_{P_{X}}=MI_{P_{XX}}$.

**Figure 4:**
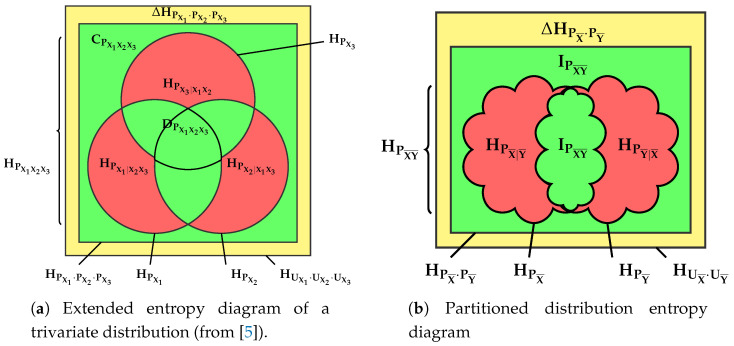
(Color online) Extended entropy diagram of multivariate distributions for (**a**) a trivariate distribution (from [5]) as an instance of Situation 1; and (**b**) a joint distribution where a partitioning of the variables is made evident (Situation 2). The color scheme follows that of Figure 2, to be explained in the text.

Since i-diagrams are a powerful tool to visualize the interaction of distributions in the bivariate case, we will also try to use them for sets of random variables. For multivariate generalizations of mutual information as seen in the i-diagrams, the following caveats apply:

- Their multivariate generalization is only warranted when signed measures of probability are considered, since it is well known that some of these “areas” can be negative, contrary to the geometric intuitions in this respect.
- We should retain the bounding rectangles that appear when considering the most entropic distributions with similar support to the ones being graphed [6]. This is the sense of the bounding rectangles in Figure 4a,b.

With great insight, the authors of [15] point out that some of the multivariate information measures stem from focusing on a particular property of the bivariate mutual information and generalizing it to the multivariate setting. The properties in question—including already stated (2) and (3)—are: The properties in question are:

$$\begin{array}{cc} MI_{P_{XY}} & =H_{P_{X}}+H_{P_{Y}}-H_{P_{XY}} \end{array}$$

$$\begin{array}{cc} MI_{P_{XY}} & =H_{P_{X}}-H_{P_{X|Y}}=H_{P_{Y}}-H_{P_{Y|X}} \end{array}$$

(9) $$\begin{array}{cc} MI_{P_{XY}} & =\sum_{x,y}P_{XY}\left(x,y\right)\log\frac{P_{XY}\left(x,y\right)}{P_{X}\left(x\right)P_{Y}\left(y\right)} \end{array}$$

Regarding the first situation of a vector of random variables $\bar{X}∼P_{\bar{X}}$, let $\Pi_{\bar{X}}=\prod_{i=1}^{n}P_{X_{i}}$ be the (jointly) independent distribution with similar marginals to $P_{\bar{X}}$. To picture this (virtual) distribution consider Figure 4a depicting an i-diagram for $\bar{X}=\left[X_{1},X_{2},X_{3}\right]$. Then, $\Pi_{\bar{X}}=P_{X_{1}}\cdot P_{X_{2}}\cdot P_{X_{3}}$ is the inner rectangle containing both green areas. The different extensions of mutual information that concentrate on different properties are:

- the total correlation [16], integration [17] or multi-information [18], which is a generalization of (2), represented by the green area outside $H_{P_{\bar{X}}}$.
  (10) $$\begin{array}{c} C_{P_{\bar{X}}}=H_{\Pi_{\bar{X}}}-H_{P_{\bar{X}}} \end{array}$$
- the dual total correlation [19,20] or interaction complexity [21] is a generalization of (3), represented by the green area inside $H_{P_{\bar{X}}}$:
  (11) $$\begin{array}{c} D_{P_{\bar{X}}}=H_{P_{\bar{X}}}-VI_{P_{\bar{X}}} \end{array}$$
- the interaction information [22], multivariate mutual information [23] or co-information [24] is the generalization of (9), the total amount of information to which all variables contribute.
  (12) $$\begin{array}{c} MI_{P_{\bar{X}}}=\sum P_{\bar{X}}\left(\bar{x}\right)\log\frac{P_{\bar{X}}\left(\bar{x}\right)}{\Pi_{\bar{X}}\left(\bar{x}\right)} \end{array}$$
  It is represented by the inner convex green area (within the dual total correlation), but note that it may in fact be negative for n>2 [25].
- the local exogenous information [15] or the bound information [26] is the addition of the total correlation and the dual total correlation:
  (13) $$\begin{array}{cc} M_{P_{\bar{X}}} & =C_{P_{\bar{X}}}+D_{P_{\bar{X}}}. \end{array}$$

Some of these generalizations of the multivariate case were used in [5,26] to develop a similar technique as the CBET, but applied to analyzing the information content of data sources. For this purpose, it was necessary to define for every random variable a residual entropy $H_{P_{X_{i}|X_{i}^{c}}}$, where $X_{i}^{c}=\bar{X}\backslash \left\{X_{i}\right\}$, which is not explained by the information provided by the other variables. We call residual information [15] or (multivariate) variation of information [11,26] the generalization of the same quantity in the bivariate case, i.e., the sum of these quantities across the set of random variables:

(14) $$\begin{array}{c} VI_{P_{\bar{X}}}=\sum_{i=1}^{n}H_{P_{X_{i}|X_{i}^{c}}}. \end{array}$$

Then, the variation of information can easily be seen to consist of the sum of the red areas in Figure 4a and amounts to information particular to each variable.

The main question regarding this issue is which, if any, of these generalizations of bivariate mutual information are adequate for an analogue of the entropy balance equations and triangles. Note that all of these generalizations consider $\bar{X}$ as a homogeneous set of variables, that is Situation 1 described at the beginning of this section, and none consider the partitioning of the variables in $\bar{X}$ into two subsets (Situation 2), for instance to distinguish between input and output ones, so the answer cannot be straightforward. This issue is clarified in Section 3.1.

## 3. Results

Our goal is now to find a decomposition of the entropies around characterizing a joint distribution $P_{\bar{X}\bar{Y}}$ between random vectors $\bar{X}$ and $\bar{Y}$ in ways analogous to those of (5) but considering multivariate input and output.

Note that it provides no advantage trying to do this on continuous distributions, as the entropic measures used are basic. Rather, what we actually capitalize on is in the outstanding existence of a balance equation between these apparently simple entropic concepts, and what their intuitive meanings afford to the problem of measuring the transfer of information in data processing tasks. As we set out to demonstrate in this section, our main results are in complete analogy to those of the binary case, but with the flavour of the multivariate case.

### 3.1. The Aggregate and Split Channel Multivariate Balance Equation

Consider the modified information diagram of Figure 4b highlighting entropies for some distributions around $P_{\bar{X}\bar{Y}}$. When we distinguish two random vectors in the set of variables $\bar{X}$ and $\bar{Y}$, a proper multivariate generalization of the variation of information in (4) is

(15) $$\begin{array}{c} VI_{P_{\bar{X}\bar{Y}}}=H_{P_{\bar{X}|\bar{Y}}}+H_{P_{\bar{Y}|\bar{X}}}\;. \end{array}$$

and we will also call it the *variation of information.* It represents the addition of the information in $\bar{X}$ not shared with $\bar{Y}$ and vice-versa, as captured by the red area in Figure 4b. Note that this is a non-negative quantity, since its is the addition of two entropies.

Next, consider

- $U_{\bar{X}\bar{Y}}$ , the uniform distribution over the supports of $\bar{X}$ and $\bar{Y}$, and
- $P_{\bar{X}}\times P_{\bar{Y}}$, the distribution created with the marginals of $P_{\bar{X}\bar{Y}}$ considered independent.

Then, we may define a *multivariate divergence with respect to uniformity*—in analogy to (1)—as

(16) $$\begin{array}{c} \Delta H_{P_{\bar{X}}\times P_{\bar{Y}}}=H_{U_{\bar{X}\bar{Y}}}-H_{P_{\bar{X}}\times P_{\bar{Y}}}\;. \end{array}$$

This is the yellow area in Figure 4b representing the divergence of the virtual distribution $P_{\bar{X}}\times P_{\bar{Y}}$ with respect to uniformity. The virtuality comes from the fact that this distribution does not properly exist in the context being studied. Rather, it only appears in the extreme situation that the marginals of $P_{\bar{X}\bar{Y}}$ are independent.

Furthermore, recall that both the total entropy of the uniform distribution and the divergence from uniformity factor into individual equalities $H_{U_{\bar{X}}U_{\bar{Y}}}=H_{U_{\bar{X}}}+H_{U_{\bar{Y}}}$—since uniform joint distributions always have independent marginals—and $H_{P_{\bar{X}}\times P_{\bar{Y}}}=H_{P_{\bar{X}}}+H_{P_{\bar{Y}}}$. Therefore (16) admits splitting as $\Delta H_{P_{\bar{X}}\times P_{\bar{Y}}}=\Delta H_{P_{\bar{X}}}+\Delta H_{P_{\bar{Y}}}$ where

(17) $$\begin{array}{cccc} \Delta H_{P_{\bar{X}}} & =H_{U_{\bar{X}}}-H_{P_{\bar{X}}} & \Delta H_{P_{\bar{Y}}} & =H_{U_{\bar{Y}}}-H_{P_{\bar{Y}}}\;. \end{array}$$

Now, both $U_{\bar{X}}$ and $U_{\bar{Y}}$ are the most entropic distributions definable in the support of $\bar{X}$ and $\bar{Y}$ whence both $\Delta H_{P_{\bar{X}}}$ and $\Delta H_{P_{\bar{Y}}}$ are non-negative, as is their addition. These generalizations are straightforward and intuitively mean that *we expect them to agree with the intuitions developed in the CBET*, which is an important usability concern.

The problem is finding a quantity that fulfills the same role as the (bivariate) mutual information. The first property that we would like to have is for this quantity to be a “transmitted information” after conditioning away any of the entropy of either partition, so we propose the following as a definition:

(18) $$\begin{array}{c} I_{P_{\bar{X}\bar{Y}}}=H_{P_{\bar{X}\bar{Y}}}-VI_{P_{\bar{X}\bar{Y}}} \end{array}$$

represented by the inner green area in the i-diagram of Figure 4b. This can easily be “refocused” on each of the subsets of the partition:

**Lemma** **1.** *Let $P_{\bar{X}\bar{Y}}$ be a discrete joint distribution. Then*

(19) $$\begin{array}{c} H_{P_{\bar{X}}}-H_{P_{\bar{X}|\bar{Y}}}=H_{P_{\bar{Y}}}-H_{P_{\bar{Y}|\bar{X}}}=I_{P_{\bar{X}\bar{Y}}} \end{array}$$

**Proof.**  Recalling that the conditional entropies are easily related to the joint entropy by the chain rule $H_{P_{\bar{X}\bar{Y}}}=H_{P_{\bar{X}}}+H_{P_{\bar{Y}|\bar{X}}}=H_{P_{\bar{Y}}}+H_{P_{\bar{X}|\bar{Y}}}$, simply subtract $VI_{P_{\bar{X}\bar{Y}}}$. ☐

This property introduces the notion that this information is *within* each of $\bar{X}$ and $\bar{Y}$ *independently but mutually induced.* It is easy to see that this quantity appears once again in the i-diagram:

**Lemma** **2.** *Let $P_{\bar{X}\bar{Y}}$ be a discrete joint distribution. Then*

(20) $$\begin{array}{c} I_{P_{\bar{X}\bar{Y}}}=H_{P_{\bar{X}}\times P_{\bar{Y}}}-H_{P_{\bar{X}\bar{Y}}}\;. \end{array}$$

**Proof.**  Considering the entropy decomposition of $P_{\bar{X}}\times P_{\bar{Y}}$:

$$\begin{array}{c} H_{P_{\bar{X}}\times P_{\bar{Y}}}-H_{P_{\bar{X}\bar{Y}}}=H_{P_{\bar{X}}}+H_{P_{\bar{Y}}}-\left(H_{P_{\bar{Y}}}+H_{P_{\bar{X}|\bar{Y}}}\right)=H_{P_{\bar{X}}}-H_{P_{\bar{X}|\bar{Y}}}=I_{P_{\bar{X}\bar{Y}}} \end{array}$$

 ☐

In other words, this is the quantity of information required to bind $P_{\bar{X}}$ and $P_{\bar{Y}}$; equivalently, it is the amount of information *lost* from $P_{\bar{X}}\times P_{\bar{Y}}$ to achieve the binding in $P_{\bar{X}\bar{Y}}$. Pictorially, this is the outermost green area in Figure 4b, and *it must be non-negative*, since $P_{\bar{X}}\times P_{\bar{Y}}$ is more entropic than $P_{\bar{X}\bar{Y}}$. Notice that (18) and (19) are the analogues of (10) and (11), respectively, but with the flavor of (2) and (3). Therefore, this quantity must be the multivariate mutual information of $P_{\bar{X}\bar{Y}}$ as per the Kullback-Leibler divergence definition:

**Lemma** **3.** *Let $P_{\bar{X}\bar{Y}}$ be a discrete joint distribution. Then*

(21) $$\begin{array}{c} I_{P_{\bar{X}\bar{Y}}}=\sum_{i,j}P_{\bar{X}\bar{Y}}\left(x_{i},y_{j}\right)\log\frac{P_{\bar{X}\bar{Y}}\left(x_{i},y_{j}\right)}{P_{\bar{X}}\left(x_{i}\right)P_{\bar{Y}}\left(y_{j}\right)} \end{array}$$

**Proof.**  This is an easy manipulation.

$$\begin{array}{cc} \sum_{i,j}P_{\bar{X}\bar{Y}}\left(x_{i},y_{j}\right)\log\frac{P_{\bar{X}\bar{Y}}\left(x_{i},y_{j}\right)}{P_{\bar{X}}\left(x_{i}\right)P_{\bar{Y}}\left(y_{j}\right)} & =\sum_{i,j}P_{\bar{X}\bar{Y}}\left(x_{i},y_{j}\right)\log\frac{P_{\bar{X}|\bar{Y}=y_{j}}\left(x_{i}|y_{j}\right)}{P_{\bar{X}}\left(x_{i}\right)}=\sum_{i}P_{\bar{X}}\left(x_{i}\right)\log\frac{1}{P_{\bar{X}}\left(x_{i}\right)}-\\ & -\sum_{j}P_{\bar{Y}}\left(y_{j}\right)\sum_{i}P_{\bar{X}|\bar{Y}=y_{j}}\left(x_{i}|y_{j}\right)\log\frac{1}{P_{\bar{X}|\bar{Y}=y_{j}}\left(x_{i}|y_{j}\right)}=\\ & =H_{P_{\bar{X}}}-H_{P_{\bar{X}|\bar{Y}}}=I_{P_{\bar{X}\bar{Y}}}, \end{array}$$

after a step of marginalization and considering (3). ☐

With these relations we can state our first theorem:

**Theorem** **1.** *Let $P_{\bar{X}\bar{Y}}$ be a discrete joint distribution. Then the following decomposition holds:*

(22) $$\begin{array}{cc} H_{U_{\bar{X}}\times U_{\bar{Y}}} & =\Delta H_{P_{\bar{X}}\times P_{\bar{Y}}}+2\cdot I_{P_{\bar{X}\bar{Y}}}+VI_{P_{\bar{X}\bar{Y}}}\\ 0 & \leq \Delta H_{P_{\bar{X}}\times P_{\bar{Y}}},I_{P_{\bar{X}\bar{Y}}},VI_{P_{\bar{X}\bar{Y}}}\leq H_{U_{\bar{X}}\times U_{\bar{Y}}} \end{array}$$

**Proof.**  From (16) we have $H_{U_{\bar{X}}\times U_{\bar{Y}}}=\Delta H_{P_{\bar{X}}\times P_{\bar{Y}}}+H_{P_{\bar{X}}\times P_{\bar{Y}}}$ whence by introducing (18) and (20) we obtain:

(23) $$\begin{array}{c} H_{U_{\bar{X}}\times U_{\bar{Y}}}=\Delta H_{P_{\bar{X}}\times P_{\bar{Y}}}+I_{P_{\bar{X}\bar{Y}}}+H_{P_{\bar{X}\bar{Y}}}=\Delta H_{P_{\bar{X}}\times P_{\bar{Y}}}+I_{P_{\bar{X}\bar{Y}}}+I_{P_{\bar{X}\bar{Y}}}+VI_{P_{\bar{X}\bar{Y}}}. \end{array}$$

Recall that each quantity is non-negative by (15), (16) and (21), so the only things left to be proven are the limits for each quantity in the decomposition. For that purpose, consider the following clarifying *conditions*,

1. **$\bar{X}$ marginal uniformity** when $H_{P_{\bar{X}}}=H_{U_{\bar{X}}}$, **$\bar{Y}$ marginal uniformity** when $H_{P_{\bar{Y}}}=H_{U_{\bar{Y}}}$ and **marginal uniformity** when both conditions coocur.
2. **Marginal independence**, when $P_{\bar{X}\bar{Y}}=P_{\bar{X}}\times P_{\bar{Y}}$.
3. **$\bar{Y}$ determines $\bar{X}$** when $H_{P_{\bar{X}|\bar{Y}}}=0$, **$\bar{X}$ determines $\bar{Y}$** when $H_{P_{\bar{Y}|\bar{X}}}=0$ and **mutual determination**, when both conditions hold.

Notice that these conditions are *independent of each other* and that *each fixes the value of one of the quantities in the balance*:

- For instance, in case $H_{P_{\bar{X}}}=H_{U_{\bar{X}}}$ then $\Delta H_{P_{\bar{X}}}=0$ after (17). Similarly, if $H_{P_{\bar{Y}}}=H_{U_{\bar{Y}}}$ then $\Delta H_{P_{\bar{Y}}}=0$. Hence when marginal uniformity holds, we have $\Delta H_{P_{\bar{X}\bar{Y}}}=0$.
- Similarly, when marginal independence holds, we see that $I_{P_{\bar{X}|\bar{Y}}}=0$ from (20). Otherwise stated, $H_{P_{\bar{X}|\bar{Y}}}=H_{P_{\bar{X}}}$ and $H_{P_{\bar{Y}|\bar{X}}}=H_{P_{\bar{Y}}}$.
- Finally, if mutual determination holds—that is to say the variables in either set are deterministic functions of those of the other set—by the definition of the multivariate variation of information, we have $VI_{P_{\bar{X}|\bar{Y}}}=0$.

Therefore, these three conditions fix the lower bounds for their respectively related quantities. Likewise, the upper bounds hold when *two* of the conditions hold at the same time. This is easily seen invoking the previously found balance Equation (23):

- For instance, if marginal uniformity holds, then $\Delta H_{P_{\bar{X}\bar{Y}}}=0$. But if marginal independence also holds, then $I_{P_{\bar{X}|\bar{Y}}}=0$ whence by (23) $VI_{P_{\bar{X}\bar{Y}}}=H_{U_{\bar{X}}\times U_{\bar{Y}}}$.
- But if both marginal uniformity and mutual determination hold, then we have $\Delta H_{P_{\bar{X}\bar{Y}}}=0$ and $VI_{P_{\bar{X}\bar{Y}}}=0$ so that $I_{P_{\bar{X}\bar{Y}}}=H_{U_{\bar{X}}\times U_{\bar{Y}}}$.
- Finally, if both mutual determination and marginal indepence holds, then a fortiori $\Delta H_{P_{\bar{X}\bar{Y}}}=H_{U_{\bar{X}}\times U_{\bar{Y}}}$.

This concludes the proof. ☐

Notice how the bounds also allow an interpretation similar to that of (5). In particular, the interpretation of the conditions for actual joint distributions will be taken again in Section 3.2.

The next question is whether the balance equation also admits splitting.

**Theorem** **2.** *Let $P_{\bar{X}\bar{Y}}$ be a discrete joint distribution. Then the Channel Multivariate Entropy Balance equation can be split as:*

(24) $$\begin{array}{cccc} H_{U_{\bar{X}}} & =\Delta H_{P_{\bar{X}}}+I_{P_{\bar{X}\bar{Y}}}+H_{P_{\bar{X}|\bar{Y}}} & 0 & \leq \Delta H_{P_{\bar{X}}},I_{P_{\bar{X}\bar{Y}}},H_{P_{\bar{X}|\bar{Y}}}\leq H_{U_{\bar{X}}} \end{array}$$

(25) $$\begin{array}{cccc} H_{U_{\bar{Y}}} & =\Delta H_{P_{\bar{Y}}}+I_{P_{\bar{X}\bar{Y}}}+H_{P_{\bar{Y}|\bar{X}}} & 0 & \leq \Delta H_{P_{\bar{Y}}},I_{P_{\bar{X}\bar{Y}}},H_{P_{\bar{Y}|\bar{X}}}\leq H_{U_{\bar{Y}}} \end{array}$$

**Proof.**  We prove (24): the proof of (25) is similar *mutatis mutandis.*

In a similar way as for (22), we have that $H_{U_{\bar{X}}}=\Delta H_{P_{\bar{X}}}+H_{P_{\bar{X}}}$. By introducing the value of $H_{P_{\bar{X}}}$ from (19) we obtain the decomposition of $H_{U_{\bar{X}}}$ of (24).

These quantities are non-negative, as mentioned. Next consider the $\bar{X}$ marginal uniformity condition applied to the input vector introduced in the proof of Theorem 1. Clearly, $\Delta H_{\bar{X}}=0$. Marginal independence, again, is the condition so that $I_{\bar{X}\bar{Y}}=0$. Finally, if $\bar{Y}$ determines $\bar{X}$ then $H_{P_{\bar{X}|\bar{Y}}}=0$. These conditions individually provide the lower bounds on each quantity.

On the other hand, when we put together any two of these conditions, we obtain the upper bound for the unspecified variable: so, if $\Delta H_{P_{\bar{X}}}=0$ and $I_{P_{\bar{X}\bar{Y}}}=0$ then $H_{P_{\bar{X}|\bar{Y}}}=H_{P_{\bar{X}}}=H_{U_{\bar{X}}}$. Also, if $I_{P_{\bar{X}\bar{Y}}}=0$ and $H_{P_{\bar{X}|\bar{Y}}}=0$, then $H_{P_{\bar{X}}}=H_{P_{\bar{X}|\bar{Y}}}=0$ and $\Delta H_{P_{\bar{X}}}=H_{U_{\bar{X}}}-0$. Finally, if $H_{P_{\bar{X}|\bar{Y}}}=0$ and $\Delta H_{P_{\bar{X}}}=0$, then $I_{P_{\bar{X}\bar{Y}}}=H_{P_{\bar{X}}}-H_{P_{\bar{X}|\bar{Y}}}=H_{U_{\bar{X}}}-0$ ☐.

### 3.2. Visualizations: From i-Diagrams to Entropy Triangles

#### 3.2.1. The Channel Multivariate Entropy Triangle

Our next goal is to develop an exploratory analysis tool similar to the CBET introduced in Section 2.1. As in that case, we need the equation of a simplex to represent the information balance of a multivariate transformation. For that purpose, as in (6) we may normalize by the overall entropy $H_{U_{\bar{X}}\times U_{\bar{Y}}}$ to obtain the equation of the 2-simplex in multivariate entropic space,

(26) $$\begin{array}{cc} 1 & =\Delta^{\prime }H_{P_{\bar{X}}\times P_{\bar{Y}}}+2\cdot I_{P_{\bar{X}\bar{Y}}}^{\prime }+VI_{P_{\bar{X}\bar{Y}}}^{\prime }\\ 0 & \leq \Delta^{\prime }H_{P_{\bar{X}}\times P_{\bar{Y}}},I_{P_{\bar{X}\bar{Y}}}^{\prime },VI_{P_{\bar{X}\bar{Y}}}^{\prime }\leq 1\;. \end{array}$$

The de Finetti diagram of this equation then provides the aggregated *Channel Multivariate Entropy Triangle, CMET*.

**Figure 5:**
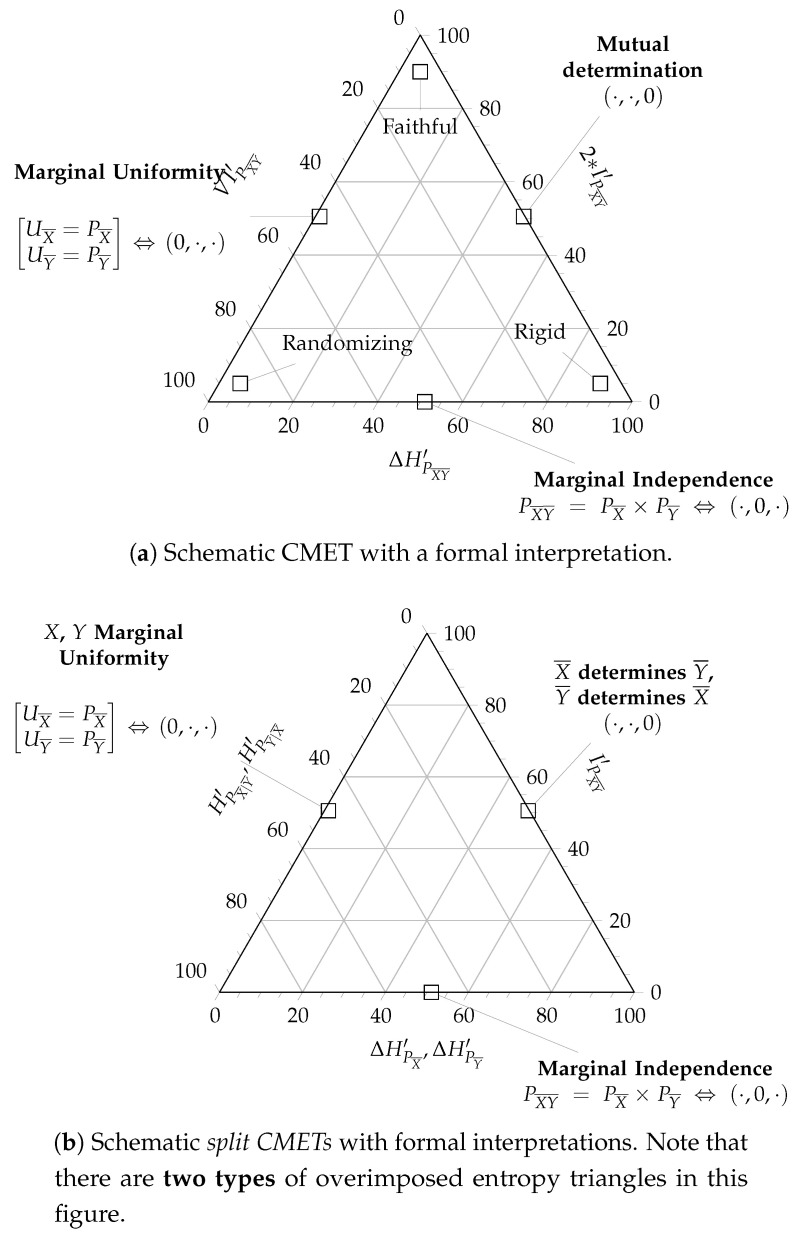
Schematic Channel Multivariate Entropy Triangles (CMET) showing interpretable zones and extreme cases using formal conditions. The annotations on the center of each side are meant to hold for that whole side, those for the vertices are meant to hold in their immediate neighborhood too.

A *formal* graphical assessment of multivariate joint distribution with the CMET is fairly simple using the schematic in Figure 5a and the conditions of Theorem 1:

- The lower side of the triangle with $I_{P_{\bar{X}\bar{Y}}}^{\prime }=0$, affected of *marginal independence* $P_{\bar{X}\bar{Y}}=P_{\bar{X}}\times P_{\bar{Y}}$, is the locus of partitioned joint distributions who do not share information between the two blocks $\bar{X}$ and $\bar{Y}$.
- The right side of the triangle with $VI_{P_{\bar{X}\bar{Y}}}^{\prime }=0$, described with *mutual determination* $H_{P_{\bar{X}|\bar{Y}}}^{\prime }=0=H_{P_{\bar{Y}|\bar{X}}}^{\prime }$, is the locus of partitioned joint distributions whose groups do not carry supplementary information to that provided by the other group.
- The left sidewith $\Delta H_{P_{\bar{X}\bar{Y}}}^{\prime }=0$, describing distributions with *uniform marginals* $P_{\bar{X}}=U_{\bar{X}}$ and $P_{\bar{Y}}=U_{\bar{Y}}$, is the locus of partitioned joint distributions that offer as much potential information for transformations as possible.

Based on these characterizations we can attach interpretations to other regions of the CMET:

- If we want a transformation from $\bar{X}$ to $\bar{Y}$ to be *faithful*, then we want to maximize the information used for mutual determination $I_{P_{\bar{X}\bar{Y}}}^{\prime }\to 1$, equivalently, minimize at the same time the divergence from uniformity $\Delta H_{P_{\bar{X}\bar{Y}}}^{\prime }\to 0$ and the information that only pertains to each of the blocks in the partition $VI_{P_{\bar{X}\bar{Y}}}^{\prime }\to 0$. So the coordinates of a faithful partitioned joint distribution will lay close to the apex of the triangle.
- However, if the coordinates of a distribution lay close to the left vertex $VI_{P_{\bar{X}\bar{Y}}}^{\prime }\to 1$, then it shows marginal uniformity $\Delta H_{P_{\bar{X}\bar{Y}}}^{\prime }\to 0$ but shares little or no information between the blocks $I_{P_{\bar{X}\bar{Y}}}^{\prime }\to 0$, hence it must be a *randomizing* transformation.
- Distributions whose coordinates lay close to the right vertex $\Delta H_{P_{\bar{X}\bar{Y}}}^{\prime }\to 1$ are essentially deterministic and in that sense carry no information $I_{P_{\bar{X}\bar{Y}}}^{\prime }\to 0,VI_{P_{\bar{X}\bar{Y}}}^{\prime }\to 0$. Indeed in this instance there does not seem to exist a transformation, whence we call them *rigid*.

These qualities are annotated on the vertices of the schematic CMET of Figure 5a. Note that different applications may call for partitioned distributions with different qualities and the one used above is pertinent when the partitioned joint distributions models a transformation of $\bar{X}$ into $\bar{Y}$ or vice-versa.

#### 3.2.2. Normalized Split Channel Multivariate Balance Equations

With a normalization similar to that from (7) to (8), (24) and (25) naturally lead to 2-simplex equations normalizing by $H_{U_{\bar{X}}}$ and $H_{U_{\bar{Y}}}$, respectively

(27) $$\begin{array}{cccc} 1 & =\Delta^{\prime }H_{P_{\bar{X}}}+I_{P_{\bar{X}\bar{Y}}}^{\prime }+H_{P_{\bar{X}|\bar{Y}}}^{\prime } & 0 & \leq \Delta^{\prime }H_{P_{\bar{X}}},I_{P_{\bar{X}\bar{Y}}}^{\prime },H_{P_{\bar{X}|\bar{Y}}}^{\prime }\leq 1 \end{array}$$

(28) $$\begin{array}{cccc} 1 & =\Delta^{\prime }H_{P_{\bar{Y}}}+I_{P_{\bar{X}\bar{Y}}}^{\prime }+H_{P_{\bar{Y}|\bar{X}}}^{\prime } & 0 & \leq \Delta^{\prime }H_{P_{\bar{Y}}},I_{P_{\bar{X}\bar{Y}}}^{\prime },H_{P_{\bar{Y}|\bar{X}}}^{\prime }\leq 1 \end{array}$$

Note that the quantities $\Delta H_{P_{\bar{X}}}^{\prime }$ and $\Delta H_{P_{\bar{Y}}}^{\prime }$ have been independently motivated and named *redundancies* ([9], Section 2.4).

These are actually two different representations for each of the two blocks in the partitioned joint distribution. Using the fact that they share one coordinate—$I_{P_{\bar{X}\bar{Y}}}^{\prime }$—and the rest are analogues—$\Delta^{\prime }H_{P_{\bar{X}}}$ and $\Delta^{\prime }H_{P_{\bar{Y}}}$ on one side, and $H_{P_{\bar{X}|\bar{Y}}}^{\prime }$ and $H_{P_{\bar{Y}|\bar{X}}}^{\prime }$ on the other—we can represent both equations *at the same time* in a single de Finetti diagram. We call this representation the *split Channel Multivariate Entropy Triangle*, an schema of which can be seen in Figure 5b. The qualifying “split” then refers to the fact that each partitioned joint distribution appears as *two points* in the diagram. Note the double annotation in the left and bottom coordinates implying that there are *two* different diagrams overlapping.

**Figure 6:**
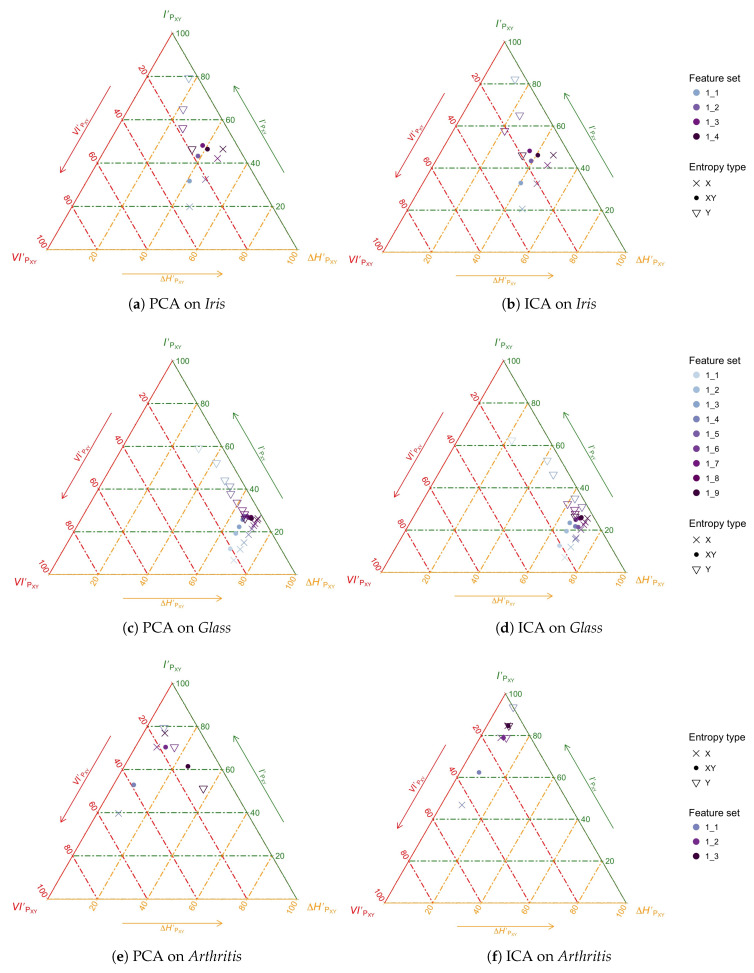
(Color online) Split CMET exploration of feature transformation and selection with PCA (left) and ICA (right) on *Iris*, *Glass* and *Arthritis* when selecting the first *n* ranked features as obtained for each method. The colors of the axes have been selected to match those of Figure 4.

Conventionally, the point referring to the $\bar{X}$ block described by (27) is represented with a cross, while the point referring to the $\bar{Y}$ block described by (28) is represented with a circle as will be noted in Figure 6.

The formal interpretation of this split diagram with the conditions of Theorem 1 follows that of the aggregated CMET but considering only one block at a time, for instance, for $\bar{X}$:

- The lower side of the triangle is interpreted as before.
- The right side of the triangle is the locus of the partitioned joint distribution whose $\bar{X}$ block is completely determined by the $\bar{Y}$ block, that is, $H_{P_{\bar{X}|\bar{Y}}}^{\prime }=0$.
- The left side of the triangle $\Delta H_{P_{\bar{X}}}^{\prime }=0$ is the locus of those partitioned joint distributions whose $\bar{X}$ marginal is uniform $P_{\bar{X}}=U_{\bar{X}}$ .

The interpretation is analogue for $\bar{Y}$ *mutatis mutandis*.

The purpose of this representation is to investigate the formal conditions separately on each block. However, for this split representation we have to take into consideration that the normalizations may not be the same, that is $H_{P_{\bar{X}}}$ and $H_{P_{\bar{Y}}}$ are, in general, different.

A full example of the interpretation of both types of diagrams, the CMET and the split CMET is provided in the next Section in the context of feature transformation and selection.

### 3.3. Example Application: The Analysis of Feature Transformation and Selection with Entropy Triangles

In this Section we present an application of the results obtained above to a machine learning subtask: the transformation and selection of features for supervised classification.

***The task.*** An extended practice in supervised classification is to explore different transformations of the observations and then evaluate such different approaches on different classifiers for a particular task [27]. Instead of this “in the loop” evaluation—that conflates the evaluation of the transformation and the classification—we will use the CMET to evaluate *only* the transformation block using the information transferred from the original to the transformed features as heuristic. As specific instances of transformations, we will evaluate the use of Principal Component Analysis (PCA) [28] and Independent Component Analysis (ICA) [29] which are often employed for dimensionality reduction.

Note that we may evaluate feature transformation and dimensionality reduction at the same time with the techniques developed above: the transformation procedure in the case of PCA and ICA may provide the $\bar{Y}$ as a ranking of features, so that we may carry out *feature selection* afterwards by selecting subsets ${\bar{Y}}_{j}$ spanning from the first-ranked to the *j*-th feature.

***The tools.*** PCA is a staple technique in statistical data analysis and machine learning based in the Singular Value Decomposition of the data matrix to obtain projections along the singular vectors that account for its variance in decreasing amount, so PCA ranks the transformed features by this order. The implementation used in our examples are those of the publicly available R packages stats (v. 3.3.3) (https://stat.ethz.ch/R-manual/R-devel/library/stats/html/00Index.html, accessed on 11 June 2018).

While PCA aims at the orthogonalization of the projections, ICA finds the projections, also known as *factors*, by maximimizing their statistical independence, in our example by minimizing a cost term related to their mutual information [30]. However, this does not result in a ranking of the transformed features, hence we have created a pseudo-ranking by carrying an ICA transformation obtaining *j* transformed features for all sensible values of 1≤j≤l using independent runs of the ICA algorithm. The implementation used in our examples is that of fastICA [30] as implemented in the R package fastICA (v. 1.2-1) (https://cran.r-project.org/package=fastICA, accessed on 11 June 2018, with standard parameter values (alg.typ=“parallel”, fun=“logcosh”, alpha=1, method=“C”, row.norm= FALSE, maxit=200, tol=0.0001).

The entropy diagrams and calculations were carried out with the open-source entropies experimental R package that provides an implementation of the present framework (available at https://github.com/FJValverde/entropies.git, accessed on 11 June 2018). The analysis carried out in this section is part of an illustrative vignette for the package and will remain so in future releases.

***Analysis of results.*** We analyzed in this way some UCI classification datasets [31], whose number of classes *k*, features *m*, and observations *n* are listed in Table 1.

For simplicity issues, we decided to illustrate our new techniques on three datasets: *Iris*, *Glass* and *Arthritis*. *Ionosphere*, *BreastCancer*, *Sonar* and *Wine* have a similar pattern to *Glass*, but less interesting, as commented below. Besides, both *Ionosphere* and *Wine* have too many features for the kind of neat visualization we are trying to use in this paper. We have also used a slightly modified entropy triangles in which the colors of the axes are related to those of the information diagrams of Figure 4b.

For instance, Figure 6a presents the results of the PCA transformation on the logarithm of the features of Anderson’s Iris. Crosses represent the information decomposition of the input features $\bar{X}$ using (27) while circles represent the information decomposition of transformed features ${\bar{Y}}_{j}$ using (28) and filled circles the aggregate decomposition of (26). We represent several possible features sets ${\bar{Y}}_{j}$ as output where each is obtained selecting the first *j* features in the ranking provided by PCA. For example, since Iris has four features we can make four different feature sets of 1 to *j* features, named in the Figure as “1_*j*”, that is, “1_1” to “1_4”. The figure then explores how the information in the whole database $\bar{X}$ is transported to different, nested candidate feature sets ${\bar{Y}}_{j}$ as per the PCA recipe: choose as many ranked features as required to increase the transmitted information.

We first notice that all the points for $\bar{X}$ lie on a line parallel to the left side of the triangle and their average transmitted information is increasing, parallel to a decrease in remanent information. Indeed, the redundancy $\Delta H_{\bar{X}}^{\prime }=\frac{\Delta H_{\bar{X}}}{H_{U_{\bar{X}}}}$ is the same regardless of the choice of ${\bar{Y}}_{j}$. The monotonic increase with the number of features selected *j* in *average transmitted information* $I_{P_{\bar{X}{\bar{Y}}_{j}}}^{\prime }=\frac{I_{P_{\bar{X}{\bar{Y}}_{j}}}}{H_{U_{\bar{X}}}}$ in (27) corresponds to the monotonic increase in absolute transmitted information $I_{P_{\bar{X}{\bar{Y}}_{j}}}$: for a given input set of features $\bar{X}$, the more output features are selected, the higher the mutual information between input and output. This is the basis of the effectiveness of the feature-selection procedure.

Regarding the points for ${\bar{Y}}_{j}$, note that the *absolute* transmitted information also appears in the *average* transmitted information (with respect to ${\bar{Y}}_{j}$) as $I_{P_{\bar{X}{\bar{Y}}_{j}}}^{\prime }=\frac{I_{P_{\bar{X}{\bar{Y}}_{j}}}}{H_{U_{{\bar{Y}}_{j}}}}$ in (28). While $I_{P_{\bar{X}{\bar{Y}}_{j}}}$ increases with *j*, as mentioned, we actually see a monotonic *decrease* in $I_{P_{\bar{X}{\bar{Y}}_{j}}}^{\prime }$. The reason for this is the rapidly increasing value of the denominator $H_{U_{{\bar{Y}}_{j}}}$ as we select more and more features.

Finally, notice how these two tendencies are conflated in the aggregate plot for the $\bar{X}{\bar{Y}}_{j}$ in Figure 6a that shows a lopsided, inverted U pattern, peaking before *j* reaches its maximum. This suggests that if we balance aggregated transmitted information against number of features selected—the complexity of the representation—in the search for a *faithful* representation, the average transmitted information is the quantity to optimize, that is, the *mutual determination* between the two feature sets.

Figure 6b presents similar results on the ICA transformation on the logarithm of the features of Anderson’s Iris with the same glyph convention as before, but with a ranking resulting from carrying the ICA method *in full* for each value of *j*. That is, we first work out ${\bar{Y}}_{1}$ which is a single component, then we calculate ${\bar{Y}}_{2}$ which the two best ICA components, and so on. The reason for this is that ICA does not rank the features it produces, so we have to create this ranking by carrying the ICA algorithm for all values of *j* to obtain each ${\bar{Y}}_{j}$. Note that the transformed features produce by PCA and ICA are, in principle, very different, but the phenomena described for PCA are also apparent here: an increase in *aggregate* transmitted information, checked by the increase of the denominator represented by $H_{U_{{\bar{Y}}_{j}}}$ which implies a decreasing transmitted information *per feature* for ${\bar{Y}}_{j}$.

**Figure 7:**
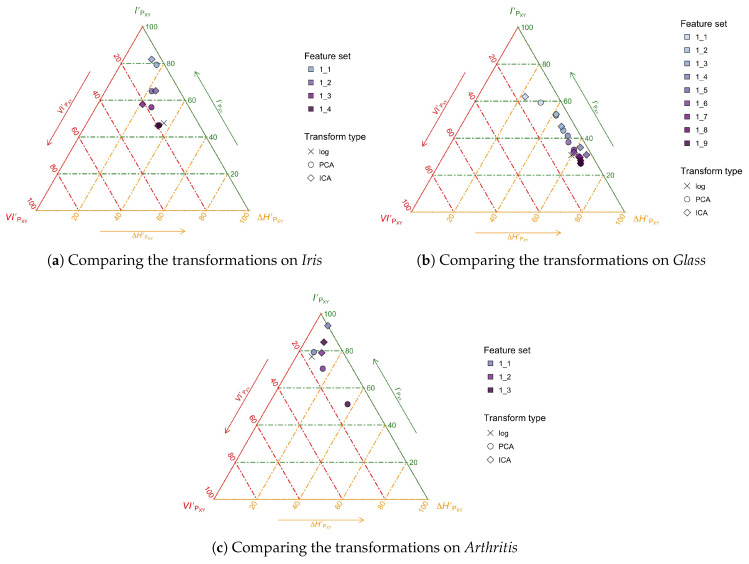
(Color online) Comparison of PCA and ICA as data transformations using the CMET on *Iris*, *Glass* and *Arthritis*. Note that these are the same positions represented as inverted triangles in Figure 6a,b.

With the present framework the question of which transformation is “better” for this dataset can be given content and rephrased as *which transformation transmits more information on average on this dataset*, and also, importantly, *whether the aggregate information available in the dataset is being transmitted* by either of these methods. This is explored in Figure 7 for *Iris*, *Glass* and *Arthritis*, where, for reference, we have included a point for the (deterministic) transformation of the logarithm, the cross, giving an idea of what a lossless information transformation can achieve.

Consider Figure 7a for *Iris*. The first interesting observation is that neither technique is transmitting all of the information in the database, which can be gleaned from the fact that both feature sets “1_4”—when all the features available have been selected—are below the cross. This clearly follows the data processing inequality, but is still surprising since transformations like ICA and PCA are extensively used and considered to work well in practice. In this instance it can only be explained by the advantages of the achieved dimensionality reduction. Actually, the observation in the CMET suggests that we can *improve on the average transmitted information per feature* by retaining the three first features for each PCA and ICA.

The analysis of *Iris* turns out to be an intermediate case between that of *Arthritis* and *Glass*, the latter being the most typical in our analysis. This is the case with a lot of original features $\bar{X}$ which transmit very little private, distinctive information per feature. The typical behavior, both for PCA and ICA is to select at first, features that carry very little average information ${\bar{Y}}_{1}$. As we select more and more transformed features, information accumulates but at a very slow pace as shown in Figure 6c,d. Typically, the transformed features chosen last are very redundant. In the case of *Glass*, specifically, there is no point in retaining features beyond the sixth (out of 9) for either PCA or ICA as shown in Figure 7b. As to comparing the techniques, in some similarly-behaving datasets PCA is better, while in others ICA is. In the case of *Glass*, it is better to use ICA when retaining up to two transformed features, but it is better to use PCA when retaining between 2 and 6.

The case of *Arthritis* is quite different, perhaps due to the small number of original features n=3. Our analyses show that just choosing the first ICA component ${\bar{Y}}_{1}$—perhaps the first two—provides an excellent characterization of the dataset, being extremely efficient in what regards information transmission. This phenomenon is also seen in the first PCA component, but is lost as we aggregate more PCA components. Crucially, taking the 3 ICA components amounts to taking all of the original information in the dataset, while taking the 3 components in the case of PCA is rather inefficient, as confirmed by Figure 7c.

All in all, our analyses show that the unsupervised transformation and selection of features in datasets can be assessed using an information-theoretical heuristic: maximize the average mutual information accumulated by the transformed features. And we have also shown how to carry out this assessment with entropic balance equations and entropy triangles.

### 3.4. Discussion

The development of the multivariate case is quite parallel to the bivariate case. An important point to realize is that the multivariate transmitted information between two different random vectors $I_{P_{\bar{X}\bar{Y}}}$ is the proper generalization for the usual mutual information $MI_{P_{XY}}$ in the bivariate case, rather than the more complex alternatives used in multivariate sources (see Section 2.2 and [5,14]). Indeed properties (18) and (20) are crucial in transporting the structure and intuitions built from the bivariate channel entropy triangle to the multivariate one, of which the former is a proper instance. This was not the case with balance equations and entropy triangles for stochastic sources of information [5].

The crucial quantities in the balance equation and the triangle have been independently motivated in other works. First, multivariate mutual information is fundamental in Information Theory, and we have already mentioned the redundancy $\Delta H_{P_{X}}$ [9]. We also mentioned the input-entropy normalized $I_{P_{\bar{X}\bar{Y}}}^{\prime }$ used as a standalone assessment measure in intrusion detection [32]. Perhaps the least known quantity in the paper was the variation of information. Despite being inspired by the concept proposed by Meila [11], to the best of our knowledge it is completely new in the multivariate setting. However, the underlying concepts of conditional or remanent entropies have proven their usefulness time and again. All of the above is indirect proof that the quantities studied in this paper are significant, and the existence of a balance equation binding them together important.

The paragraph above notwithstanding, there are researchers who claim that Shannon-type relations cannot capture all the dependencies inside multivariate random vectors [33]. Due to the novelty of that work, it is not clear how much the “standard” theory of Shannon measures would have to change to accommodate the objections raised to it in that respect. But this question seems to be off the mark for our purposes: the framework of channel balance equations and entropy triangles has not been developed to look into the question of dependency, but of *aggregate information transfer*, wherever that information comes from. It may be relevant to source balance equations and triangles [5]—which have a different purpose—but that still has to be researched into.

The normalizations involved in (6) and (26)—respectively, (8), (27) and (28)—are similar conceptually: to divide by the logarithm of the total size of the domains involved whether it is the size of X×Y or that of $\bar{X}\times \bar{Y}$. Notice, first, that this is the same as taking the logarithm base these sizes in the non-normalized equations. The resulting units would not be bits for the multivariate case proper, since the size of $\bar{X}$ or $\bar{Y}$ is at least 2×2=4. But since the entropy triangles represent compositions [12], which are inherently dimensionless, this allows us to represent many different, and otherwise incomparable systems, e.g., univariate and multivariate ones with the same kind of diagram. Second, this type of normalization allows for an interpretation of the extension of these measures to the continuous case as a limit in the process of equipartitioning a compact support, as done, for instance, for the Rényi entropy in ([34], Section 3) which is known to be a generalization of Shannon’s. There are hopes, then for a continuous version of the balance equations for Renyi’s entropy.

Finally, note that the application presented in Section 3.3 above, although principled in the framework presented here, is not conclusive on the quality of the analyzed transformations in general but only as applied to the particular dataset. For that, a wider selection of data transformation approaches, and many more datasets should be assessed. Furthermore, the feature selection process used the “filter” approach which for supervised tasks seems suboptimal. Future work will address this issue as well as how the technique developed here relates to the end-to-end assessment presented in [4] and the source characterization technique of [5].

## 4. Conclusions

In this paper, we have introduced a new way to assess quantitatively and visually the transfer of information from a multivariate source $\bar{X}$ to a multivariate sink of information $\bar{Y}$, using a heretofore unknown decomposition of the entropies around the joint distribution $P_{\bar{X}\bar{Y}}$. For that purpose, we have generalized a similar previous theory and visualization tools for bivariate sources, greatly extending the applicability of the results:

- We have been able to decompose the information of a random multivariate source into three components: (a) the non-transferable divergence from uniformity $\Delta H_{P_{\bar{X}\bar{Y}}}$, which is an entropy “missing” from $P_{\bar{X}\bar{Y}}$; (b) a transferable, but not transferred part, the variation of information $VI_{P_{\bar{X}\bar{Y}}}$; and (c) the transferable and transferred information $I_{P_{\bar{X}\bar{Y}}}$, which is a known, but never considered in this context, generalization of bivariate mutual information.
- Using the same principles as in previous developments, we have been able to obtain a new type of visualization diagram for this balance of information using de Finetti’s ternary diagrams, which is actually an exploratory data analysis tool.

We have also shown how to apply these new theoretical developments and the visualization tools to the analysis of information transfer in unsupervised feature transformation and selection, a ubiquitous step in data analysis, and specifically, to apply it to the analysis of PCA and ICA. We believe this is a fruitful approach, e.g., for the assessment of learning systems, and foresee a bevy of applications to come. Further conclusions on this issue are left for a more thorough later investigation.

The authors declare no conflict of interest.

## Figures and Tables

**Table 1:** Datasets analyzed.

	Name	k	m	n
1	Ionosphere	2	34	351
2	**Iris**	3	4	150
3	**Glass**	7	9	214
4	**Arthritis**	3	3	84
5	BreastCancer	2	9	699
6	Sonar	2	60	208
7	Wine	3	13	178

## Abbreviations

PCA	Principal Component Analysis
ICA	Independent Component Analysis
CMET	Channel Multivariate Entropy Triangle
CBET	Channel Binary Entropy Triangle
SMET	Source Multivariate Entropy Triangle
